# The Class IIA Histone Deacetylase (HDAC) Inhibitor TMP269 Downregulates Ribosomal Proteins and Has Anti-Proliferative and Pro-Apoptotic Effects on AML Cells

**DOI:** 10.3390/cancers15041039

**Published:** 2023-02-07

**Authors:** Laura Urwanisch, Michael Stefan Unger, Helene Sieberer, Hieu-Hoa Dang, Theresa Neuper, Christof Regl, Julia Vetter, Susanne Schaller, Stephan M. Winkler, Emanuela Kerschbamer, Christian X. Weichenberger, Peter W. Krenn, Michela Luciano, Lisa Pleyer, Richard Greil, Christian G. Huber, Fritz Aberger, Jutta Horejs-Hoeck

**Affiliations:** 1 Department of Biosciences and Medical Biology, University of Salzburg, 5020 Salzburg, Austria; 2 Cancer Cluster Salzburg (CCS), 5020 Salzburg, Austria; 3 Bioinformatics Research Group, University of Applied Sciences Upper Austria, Softwarepark 11, 4232 Hagenberg im Muehlkreis, Austria; 4 Institute for Biomedicine, Eurac Research, Affiliated Institute of the University of Lübeck, Via A. Volta 21, 39100 Bolzano, Italy; 5 IIIrd Medical Department with Hematology and Medical Oncology, Hemostaseology, Rheumatology and Infectious Diseases, Oncologic Center, Paracelsus Medical University, 5020 Salzburg, Austria; 6 Salzburg Cancer Research Institute with Laboratory of Immunological and Molecular Cancer Research and Center for Clinical Cancer and Immunology Trials, 5020 Salzburg, Austria

**Keywords:** AML, HDAC, HDAC inhibitor, TMP269, RPL6, proliferation, venetoclax, azacitidine, apoptosis

## Abstract

**Simple Summary:**

Epigenetic alterations strongly contribute to the development of various types of cancer, including blood cancers such as acute myeloid leukemia (AML). Unlike genetic mutations, epigenetic changes in cancer are reversible. Therefore, targeted modification of epigenetic regulators, such as histone deacetylases (HDACs), is under intense investigation. Here, we analyzed gene expression for all four classes of HDACs in AML patients compared to healthy controls. We observed significant overexpression of various HDACs, including the relatively unexplored HDAC class IIA members. To investigate the cellular consequences of HDAC inhibition, we treated AML cell lines with the class IIA HDAC inhibitor TMP269 and observed significant effects on the cellular proteome and the growth of AML cells. We further demonstrate that the combination of TMP269 and venetoclax results in enhanced cell apoptosis. Our work provides new data for the HDAC inhibitor TMP269 and suggests that TMP269 might be an alternative compound for polytherapy in AML.

**Abstract:**

Acute myeloid leukemia (AML) is a hematopoietic malignancy characterized by altered myeloid progenitor cell proliferation and differentiation. As in many other cancers, epigenetic transcriptional repressors such as histone deacetylases (HDACs) are dysregulated in AML. Here, we investigated (1) HDAC gene expression in AML patients and in different AML cell lines and (2) the effect of treating AML cells with the specific class IIA HDAC inhibitor TMP269, by applying proteomic and comparative bioinformatic analyses. We also analyzed cell proliferation, apoptosis, and the cell-killing capacities of TMP269 in combination with venetoclax compared to azacitidine plus venetoclax, by flow cytometry. Our results demonstrate significantly overexpressed class I and class II HDAC genes in AML patients, a phenotype which is conserved in AML cell lines. In AML MOLM-13 cells, TMP269 treatment downregulated a set of ribosomal proteins which are overexpressed in AML patients at the transcriptional level. TMP269 showed anti-proliferative effects and induced additive apoptotic effects in combination with venetoclax. We conclude that TMP269 exerts anti-leukemic activity when combined with venetoclax and has potential as a therapeutic drug in AML.

## 1. Introduction

Acute myeloid leukemia (AML) is one of the most common malignant diseases of the hematopoietic system in adults, with a median age at diagnosis of 68 years [1]. AML is characterized by the altered proliferation and differentiation blockade of myeloid progenitor cells, which leads to the replacement of functional blood cells by non-functional leukemic blasts, resulting in a severely impaired hematopoietic system [2]. For several decades, the standard treatment for AML included high-dosage chemotherapy with or without subsequent allogeneic stem cell transplantation according to risk profile, age, and comorbidity as well as availability of adequate donors, or various low-dose palliative treatment regimens for patients incapable of withstanding such an aggressive treatment. Since 2017, several new drugs have emerged that revolutionized the treatment of AML [3,4]. One of the new promising drugs for patients not qualifying for high-dose chemotherapy is venetoclax. FDA approved since 2018, venetoclax induces rapid apoptosis of AML cells by inhibiting the anti-apoptotic protein B cell leukemia/lymphoma-2 (BCL-2). Venetoclax shows low efficacy as a monotherapy but is highly potent when used in combination with hypomethylating agents (HMAs) such as azacitidine and decitabine or chemotherapeutic agents such as cytarabine, resulting in synergistic anti-leukemic activity [5,6]. Even though the combination therapies of HMAs and venetoclax show a high success rate, clinical studies revealed that 1/3 of patients with newly diagnosed AML do not go into complete remission and/or become resistant to the drug treatment over time [7,8]. In particular, AML patients with monocytic differentiation (M5 AML according to the French-American-British (FAB) classification system) show little or no response to the combination of azacitidine and venetoclax [9]. Therefore, there is a strong need for new combination approaches with venetoclax to improve treatment efficacy.

Histone deacetylases (HDACs) are enzymes that regulate gene expression by changing the acetylation state of N-terminal lysine residues of core histone proteins and of some non-histone proteins, including oncogenes, tumor suppressor genes, transcription factors, chaperones, and other cell signaling molecules, thereby altering protein stability or biological function [10,11]. In many cancer types, the cellular acetylation homeostasis of the proteome is impaired due to dysregulation of HDACs [12,13], resulting in altered levels of cell proliferation, cell differentiation, and apoptosis [14]. These tumor-promoting effects can be antagonized by HDAC inhibitors, which block histone deacetylation and thereby inhibit cell proliferation and migration [15,16] and induce cell-cycle arrest [17] and apoptosis [18] of cancer cells. There are different types of HDAC inhibitors which are chemically grouped as hydroximates, benzamides, cyclic peptides, and aliphatic acids, and directly or indirectly block the active site pockets of HDACs; for example, hydroximates directly bind to the cofactor zinc [19,20]. HDAC inhibitors have already been shown to exhibit anti-tumor activity in vitro and in vivo in various types of cancer [21], including AML [20]. Similarly, non-selective inhibitors targeting all classes of HDACs such as vorinostat [22,23], panobinostat [24], and trichostatin A [25,26] as well as the class I selective inhibitor MS-275 (entinostat) [27,28] have been effective against AML cell lines, but often showed only limited anti-leukemic activity in clinical and preclinical trials, especially if used as monotherapy. Currently, little is known about the molecular mechanism and therapeutic effects of specific class IIA HDAC inhibitors and their combination with venetoclax in the context of AML [29].

In this study, we describe novel effects of the specific class IIA HDAC inhibitor TMP269 [30] on AML cell lines. We performed a proteomic and comparative bioinformatic analysis of AML cells treated with TMP269 and identified the specific downregulation of a set of proteins which are upregulated in AML patients compared to healthy controls at the gene expression level. Among the most significantly downregulated proteins are ribosomal proteins (RPs), annotated as active components of cytosolic ribosomes and functionally linked to the initiation of ribosomal protein translation. As a consequence, we demonstrate that TMP269 treatment dampens AML cell growth and cell proliferation. Strikingly, we provide evidence that the combination of TMP269 and venetoclax has additive apoptosis-inducing effects on AML cells in vitro.

We propose that TMP269 mediates its anti-leukemic effects by downregulation of cancer-associated RPs, which synergize with the anti-leukemic/pro-apoptotic effects of venetoclax. Based on our results, TMP269 in combination with venetoclax may provide a novel and potent polytherapy option for AML patients.

## 2. Materials and Methods

### 2.1. Chemicals

TMP269, BML-210, and venetoclax were obtained from eubio (Vienna, Austria). MS-275 (entinostat) and bufexamac were purchased from Biozol (Eching, Germany). Azacitidine was obtained from Selleckchem (Houston, TX, USA). All compounds were dissolved in 100% DMSO and stored at −70 °C.

### 2.2. Cell Lines and Culture Conditions

The human AML cell lines KG-1a, HL-60, OCI-AML3, MOLM-13, MOLM-14, and MV4-11 were cultured in RPMI 1640 medium (KG-1a, HL-60, MOLM-13, MOLM-14), Iscove’s Modified Dulbecco’s Medium (IMDM) (MV4-11), or Minimum Essential Medium Eagle (MEM) (OCI-AML3), supplemented with 10% (HL-60, MOLM-13, MOLM-14, MV4-11) or 20% (KG-1a, OCI-AML3) heat-inactivated FBS, 1% L-glutamine, 1% penicillin, and streptomycin. Cells were kept at 37 °C in a humidified incubator with 5% CO_2_. All cell lines were obtained from the Leibniz-Institute DSMZ GmbH (https://www.dsmz.de accessed on 22 November 2022) with the following accession numbers: MOLM-13 (ACC 554), MOLM-14 (ACC 777), MV4-11 (ACC 102), HL-60 (ACC 3), OCI-AML3 (ACC 852), KG-1a (ACC 421).

### 2.3. Quantitative Real-Time PCR (RT-PCR)

Total RNA of cultured cells was extracted using TRI Reagent (Sigma, St. Louis, MO, USA) according to the manufacturer’s instructions, and reverse-transcribed into complementary DNA (cDNA) with RevertAid H Minus M-MulV reverse transcriptase (Thermo Fisher Scientific, Waltham, MA, USA). RT-PCR was performed with Luna^®^ Universal Probe qPCR Master Mix (New England BioLabs^®^ Inc., Ipswich, MA, USA) on the Rotor-Gene 3000 (Qiagen Instruments, Hombrechtikon, Switzerland). The amount of mRNA was normalized to the reference gene large ribosomal protein P0 (RPLP0). The relative mRNA expression was determined by calculating the difference between the cycle threshold of the gene of interest and the reference gene (Δ*CT*). The values are depicted as 2^−ΔCT^. The following primers (Sigma) were used: HDAC1: forward 5′- CTATCGCCCTCACAAAGCCAATGC -3′, reverse 5′- CAGCACTTGCCACAGAACCACCAG -3′; HDAC2: forward 5′- TGGACCCATCACCCAAGCAGTG -3′, reverse 5′- CCACAGGGAGGATGCCAGAACA -3′; HDAC3: forward 5′- TCGTGCTGGGTGGTGGTGGTTA -3′, reverse 5′- GATGCGGGTGCTGACATCTGGA-3′; HDAC8: forward 5′- ACGGCTCGATGCTGGACATACTTG -3′, reverse 5′- GTTGAGGATTTGTTGGATTCGGTGG -3′; HDAC4: forward 5′- CCCTGCAAATGGATGGCTTGTG -3′, reverse 5′- TGGAGACGGGAGCGGTTCTGTTA -3′; HDAC5: forward 5′- CGAAGTCAAAGGAGCCCACACCA -3′, reverse 5′- CCAGGCAAAGGCAGTTTGTAGGAG -3′; HDAC7: forward 5′- GTAGCAGCACGCCCGCATCA -3′, reverse 5′- AGCAAGGACACTGTCGGCAAGG -3′; HDAC9: forward 5′- GCGGTTGGCATGGATGGATTAGA -3′, reverse 5′- TGCTCAGGGTGGGTGGTGGAA -3′; HDAC6: forward 5′- ACTCATACTCCTGTGCCTGCCTGG -3′, reverse 5′- GCGGTGTTTCTGTTGAGCATAGCG -3′; HDAC10: forward 5′- GCCCTAGAGTCCATCCAGAGTGCC-3′, reverse 5′- GCAACAGCGGTGCGGACAGAG-3′; RPLP0: forward 5’- GGCACCATTGAAATCCTGAGTGATGTG -3’, and reverse 5’- TTGCGGACACCCTCCAGGAAG -3’.

### 2.4. Cell Proliferation Analysis

Cells were labeled with a cell proliferation dye (eBioscience™ Cell Proliferation Dye eFluor™ 450, Invitrogen™, Waltham, MA, USA) diluted 1:5000 in PBS (final concentration: 2 µM). Then, 1 × 10^5^ cells were seeded in 500 µL medium in a 48-well plate and treated with the desired concentrations of the appropriate HDAC inhibitors for 48 h. After harvesting, cells were manually counted using a Neubauer chamber and proliferation was assayed by flow cytometry on a BD FACS Canto II (BD Biosciences, San Jose, CA, USA). Fixable viability dye eFluor780 (eBioscience) was used at 1:2000 to exclude dead cells. Results were analyzed using FlowJo analysis software (BD Biosciences).

### 2.5. Apoptosis Assay

Apoptosis was determined by staining the cells with Annexin V Apoptosis Detection Kit eFluor-450 and 7-AAD (eBioscience) according to the manufacturer’s instructions. For titration of single drugs, MOLM-13 cells were treated with the indicated concentrations of TMP269, azacitidine, or venetoclax for 24 h. For combinatory treatments, MOLM-13 or HL-60 cells were treated with 12.5 µM TMP269, 1 µM azacitidine, 25 nM venetoclax, or a combination thereof (TMP269 + venetoclax or azacitidine + venetoclax) for 24 h. Further, MOLM-13 cells were treated with 5 µM BML-210, 25 nM venetoclax, or a combination thereof (BML-210 + venetoclax) for 24 h. Cell viability and apoptosis were assessed by flow cytometry using a FACS Canto II flow cytometer (BD Biosciences) and the data were analyzed using FlowJo Software (BD Biosciences).

### 2.6. LDH Assay

MOLM-13 cells were treated with different concentrations of HDAC inhibitors for 48 h. Cytotoxicity was assessed by quantification of lactate dehydrogenase (LDH) release into cell supernatant by measuring the decrease in NADH absorbance at 340 nm. As a positive control, cells were lysed with 0.10% (*v/v*) Triton X-100 for 30 min prior to harvesting to achieve maximum LDH release.

### 2.7. Database Analysis

The public genome dataset GSE13159 from NCBI’s Gene Expression Omnibus (NCBI-GEO) was used to screen all HDAC classes and to compare the *HDAC1*, *HDAC2*, *HDAC3*, *HDAC8*, *HDAC4*, *HDAC5*, *HDAC7*, *HDAC9*, *HDAC6*, *HDAC10*, *SIRT1*, *SIRT2*, *SIRT3*, *SIRT5*, *SIRT6*, *SIRT7,* and *HDAC11* expression in AML patients to that in healthy individuals. In addition to HDACs, gene expression analysis of various other genes was performed. The dataset used is part of the MILE (Microarray Innovations in Leukemia) study research program and comprises whole-genome analysis data from 542 AML patients and 74 healthy donors of a total sample size of 2096 from 11 participating centers on three continents [31,32]. In all our analyses, we used only data from bone marrow samples. Datasets from the GEO database were imported using GEOparse (https://geoparse.readthedocs.io/ accessed on 12 December 2022) and the analysis was performed using Python.

### 2.8. Proteomics

Three batches of 1 × 10^6^ MOLM-13 cells were treated with 12.5 µM TMP269 for 24 h. To analyze the proteome of the differentially treated MOLM-13 cells, label-free quantification (LFQ) was conducted. In short, the cells were lysed followed by proteolysis with trypsin and clean-up of the generated peptides by suspension trapping in an S-trap™ column (Protifi, NY, USA). Subsequently, the peptides were separated in a reversed-phase high-performance liquid chromatography system coupled to a quadrupole Orbitrap mass spectrometer. Peptide spectra were acquired in data-dependent mode, whereby each scan cycle consisted of a full scan at a resolution setting of 70,000 at *m/z* 200, followed by 15 higher-energy collisional dissociation scans at 32% normalized collision energy at a resolution setting of 17,500 at *m/z* 200. The generated peptide spectra were processed utilizing MaxQuant 2.0.1.0 [33] with default settings for LFQ. For protein identification, a database from the Uniprot consortium [34] including only reviewed Swiss-Prot entries for *Homo sapiens* (Human) from 3 February 2022 was used applying a 1% false discovery rate and a reversed sequence decoy database. The obtained protein groups were further processed using the Perseus software platform [35]. More details are described in Supplementary Materials.

### 2.9. Bioinformatics

The identified protein groups were filtered to remove decoy hits as well as proteins that were only identified by site. Next, the LFQ intensities were log2-transformed and normalized by subtraction of the median. Protein abundances yielded by MaxQuant are shown as LFQ intensities, thus removing protein groups flagged as potential contaminants or as a hit to the reverse database [36]. Furthermore, no missing intensity measurements for any of the DMSO or the TMP269 replicates were required, and the associated UniProt accession numbers were proven to be valid. Protein differential abundance analysis was carried out with NormalyzerDE web interface v 1.14.0 [37]. After an initial round of MaxQuant protein intensity normalization methods evaluation, log2-transformed MaxQuant LFQ intensities were used as input for differential expression analysis, comparing the effect of cells treated with TMP269 to DMSO-treated controls. Here, the empirical Bayes approach Limma without covariates was chosen. The false discovery rate correction was performed according to Benjamini–Hochberg [38] and proteins were classified as differentially abundant if the so-corrected *p*-values were below 0.05. Proteins reported by MaxQuant were identified by UniProt accession numbers, and the corresponding gene symbols were derived by annotation mapping provided by the Ensembl database (release 107). Only proteins with a unique mapping were kept in the dataset. Principal component analysis was visualized with the plot_pca function from the DEP R package (v 1.18.0) [39] on variance stabilizing the transformed LFQ intensity data. Functional annotation was performed with clusterProfiler (v 4.4.4) [40]; enrichment representations were visualized with the barplot function called by clusterProfiler. Gene ontology terms were considered significantly enriched if the Benjamini–Hochberg adjusted *p*-value was less than 0.05. All analyses were performed in R (v 4.2.0) and Bioconductor (v 3.15). The String database (https://string-db.org/ accessed on 6 September 2022) was used to search for protein networks and physical protein interactions.

### 2.10. Western Blot Analysis

Western blots were prepared as previously described [41]. Cell lysates were prepared in NP40 sample buffer, containing 150 mM NaCl, 1% Triton X, 50 mM Tris pH 7.4, and 1 mM PMSF (protease inhibitor) and phosphatase inhibitor, diluted 1:4 with 4X Laemmli sample buffer (Bio-Rad, Vienna, Austria) containing 10% beta-mercaptoethanol. Samples were then separated on a 4–12% NuPAGE Bis-Tris gel (Invitrogen, Vienna, Austria) and transferred onto a nitrocellulose membrane (0.45 µm). After blocking of non-specific binding sites with 5% skim milk for 1 h at room temperature (RT) under gentle agitation, the membrane was incubated with the appropriate primary antibody, prepared in 5% bovine serum albumin diluted in Tris-buffered saline containing 0.1% TWEEN20 (TBS-T) or 5% skim milk in TBS-T, overnight at 4 °C under gentle agitation. The membrane was then washed and incubated with the appropriate secondary antibody conjugated with horseradish peroxidase (HRP) for 1 h at RT under gentle agitation. After washing, the membrane was incubated with West Pico PLUS chemiluminescent substrate (Thermo Fisher Scientific), and detection was performed with a ChemiDoc Imager (Bio-Rad). The following primary antibodies were used according to the manufacturer’s instructions: anti-Acetyl-Histone H3 (Lys9) (C5B11) Rabbit mAb (#9649), anti-Histone H3 (D1H2) XP Rabbit mAb (#4499), and anti-β-Actin (13E5) Rabbit mAb (#4970, all from Cell Signaling Technology, Frankfurt, Germany). Quantification of protein levels from Western blots was performed with ImageJ and normalized to β-actin.

### 2.11. Statistical Analysis

Statistical analyses were performed with GraphPad Prism 9 software. Comparisons of multiple groups were analyzed by one-way ANOVA with Tukey’s multiple comparisons test. For comparison of two groups, the Mann–Whitney U test was used for non-parametric data. Data from at least three experiments are shown as mean ± standard deviation (SD) or as indicated in the respective figure legend. *p*-values < 0.05 were considered significant (* *p*  <  0.05, ** *p*  <  0.01, *** *p*  <  0.001, **** *p* ≤ 0.0001, ns = not significant).

## 3. Results

### 3.1. Increased Gene Expression of Specific Class I, Class IIA, and Class IIB HDAC Genes in AML Patients and Human AML Cell Lines

**Figure 1:**
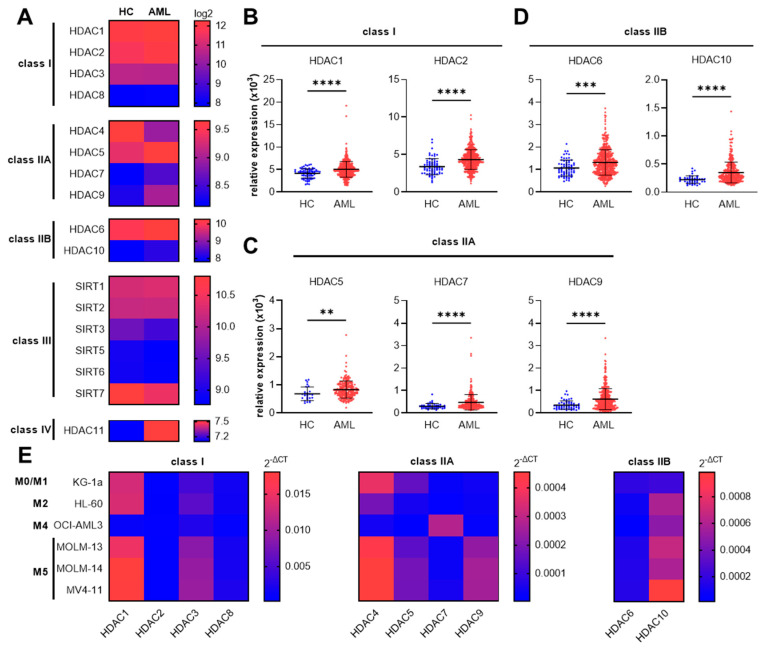
HDAC gene expression in AML patients and AML cell lines. Specific class I and class II HDAC genes are overexpressed in AML patients. (**A**) Heat map (median) of genomic database analysis for class I–IV HDAC gene expression in AML patients (AML) compared to healthy controls (HC). Data are from the MILE study (Gene Expression Omnibus—GEO: GSE13159). Significantly overexpressed HDAC genes in AML patients compared to healthy controls included *HDAC1* and *HDAC2* for class I (**B**), *HDAC5*, *HDAC7,* and *HDAC9* for class IIA (**C**), and *HDAC6* and *HDAC10* for class IIB (**D**). The dataset was imported using GEOparse from Python. Data are mean ± SD and were statistically analyzed by two-tailed Mann–Whitney U test, ** *p* ≤ 0.01, *** *p* ≤ 0.001, **** *p* ≤ 0.0001. (**E**) Heat map of relative mRNA expression (2^−ΔCT^) of class I, class IIA, and class IIB HDAC gene members in different AML cell lines measured by RT-PCR (median of n = 4–5). Red color indicates high expression and blue color indicates low expression relative to the *RPLP0* housekeeping gene.

Dysregulation of HDACs is described as a hallmark in various cancer types [13]. To investigate which classes of HDACs are predominantly dysregulated in AML patients, we first screened the gene expression of all four classes of HDACs in AML patients compared to healthy controls (Figure 1A). We used the public dataset GSE13159 from NCBI’s Gene Expression Omnibus (NCBI-GEO). This dataset is part of the MILE (Microarray Innovations in Leukemia) study research program and comprises the whole-genome expression data of bone marrow and blood samples from 542 AML patients and 74 healthy donors [31,32]. For this analysis, only bone marrow samples were used and samples from peripheral blood were excluded. We observed significantly increased HDAC gene expression in samples from AML patients compared to healthy controls for *HDAC1* and *HDAC2* (class I HDACs, Figure 1B), as well as *HDAC5*, *HDAC7,* and *HDAC9* (class IIA HDACs, Figure 1C), and *HDAC6* and *HDAC10* (class IIB HDACs, Figure 1D). Additionally, the gene expression of *HDAC4* (class IIA) and of *SIRT3*, *SIRT5*, *SIRT6*, and *SIRT7* (class III) were significantly reduced and *HDAC11* (class IV) was significantly increased in AML patients compared to healthy controls (Supplementary Figure S1A).

Having shown that several class I and class IIA/IIB HDAC genes are overexpressed in AML patient samples, we sequentially aimed to investigate whether enhanced class I and class II HDAC gene expression can also be observed in different human AML cell lines. Thus, we monitored the class I and class II HDAC mRNA expression in six AML cell lines derived from patients of different FAB subtypes (M0–M5) (Figure 1E). While most of the tested HDAC genes are expressed in all AML cell lines, the expression levels of *HDAC1* and *HDAC3* (class I HDACs) but also *HDAC4*, *HDAC5,* and *HDAC9* (class IIA HDACs), and *HDAC10* (class IIB HDAC) are highly expressed in MOLM-13, MOLM-14, as well as in MV4-11 cells. All of these cell types are representative of the M5 FAB group, which is known to be the most aggressive AML subtype [42]. Therefore, for the experiments below, the MOLM-13 cell line was selected as a representative of the M5 AML subgroup and the HL-60 cell line was chosen as a representative of the M2 subgroup, which is the most abundant subtype in AML patients [43].

Taken together, these first results identify the increased HDAC gene expression of specific class I and class II HDACs in primary AML patient samples, an expression profile which is also retained in AML patient-derived cell lines.

### 3.2. TMP269 Treatment Downregulates Ribosomal Proteins Which Are Increased in AML Patients at the Gene Expression Level

Different HDAC inhibitors have been investigated in AML [20]. One of the best-studied class I HDAC inhibitors is MS-275, also known as entinostat [44,45]. MS-275 itself and in combination with other drugs exerts antitumor activity in vitro and in vivo, as demonstrated in different tumor models, including models for AML [27,46,47,48]. In contrast, data on the function of class IIA HDACs, as well as information on the molecular mechanisms and therapeutic potential of class IIA HDAC inhibitors, are scarce. Therefore, in this study we focused on the novel selective class IIA HDAC inhibitor TMP269 [49] and its effects on different AML cell lines. TMP269 was already reported to have modest growth inhibitory effects and to enhance endoplasmic reticulum (ER) stress-mediated apoptosis in multiple myeloma cell lines in combination with the protease inhibitor carfilzomib [50].

To better understand the molecular consequences of TMP269 administration, we performed differential proteomic analysis of MOLM-13 cells treated with 12.5 µM TMP269 or DMSO (solvent control, 0.1% as final concentration) for 24 h. The concentration of 12.5 µM TMP269 was chosen based on recently published data highlighting that >10 µM TMP269 produces strong inhibition of class IIA HDAC enzyme activity in a cell-free system [30] and anti-proliferative effects in, for example, urothelial carcinoma cell lines [51].

Our proteomic analysis generated a MaxQuant output file containing an initial 3647 protein groups. After removing those flagged as potential contaminants or as hits to the reverse database, as well as protein groups with missing abundance measurements, and filtering for valid UniProt accession numbers, 2025 protein groups remained for differential abundance analysis. The further removal of 49 protein groups matching more than a single gene and quantified proteins associated to multiple Uniprot IDs gave us a final set of 1976 proteins/genes for downstream analysis. This set contained 20 upregulated and 24 downregulated proteins/genes (significant at an adjusted *p*-value < 0.05 and no limitations on expression fold change).

**Figure 2:**
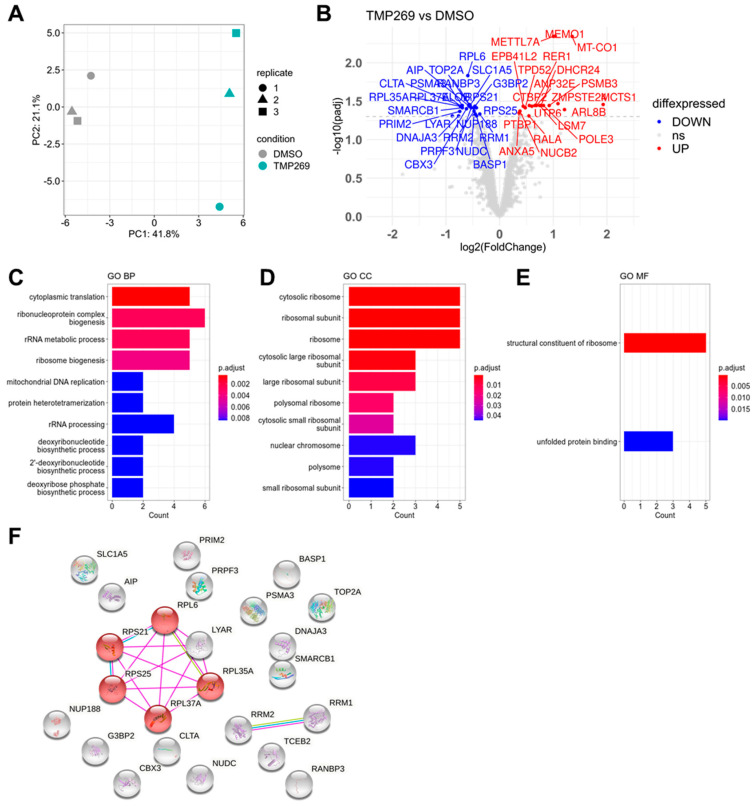
Proteomics and functional annotation analysis. TMP269 treatment downregulates ribosomal proteins. MOLM-13 cells were treated with 12.5 µM TMP269. (**A**) Principal component analysis of TMP269 and DMSO groups. (**B**) Volcano plot of significantly upregulated (red) and downregulated (blue) proteins. Gene ontology (GO) analysis of significantly downregulated proteins showing the top 10 GO terms for biological processes (BP) (**C**), cellular components (CC) (**D**), and molecular functions (MF) (**E**). (**F**) String analysis of all proteins significantly downregulated by TMP269 treatment filtered for physical protein interactions. Red highlights ribosome-associated proteins RPS21, RPL6, RPL35A, RPL37A, and RPS25 (KEGG pathway for *Ribosome*).

Principal component analysis shows a clear separation of the replicates for the TMP269 or the DMSO treatment groups (Figure 2A). Forty-four proteins were significantly differentially expressed in TMP269-treated MOLM-13 cells compared to DMSO-treated cells (Figure 2B). According to their log2-fold changes, the top upregulated protein in TMP269-treated MOLM-13 cells was Malignant T-cell-amplified Sequence 1 (MCTS1), and the top downregulated protein was cell growth-regulating nucleolar protein (LYAR). The most significantly (adjusted *p*-value < 0.05) upregulated protein was mediator of cell motility 1 (MEMO1) and the most significantly downregulated protein was 60S ribosomal protein L6 (RPL6).

Subsequently, we focused on proteins significantly downregulated by TMP269 treatment and performed gene ontology (GO) enrichment analysis for the biological process (BP), cellular component (CC), and molecular function (MF). Our analysis revealed highly enriched GO terms for ribonucleoprotein complex biogenesis and cytoplasmic translation (Figure 2C), cytosolic ribosome (Figure 2D), and structural constituent of ribosome (Figure 2E). A complete list of all significantly differentially expressed proteins together with the full GO analysis is shown in Supplementary File S1. We entered the significantly downregulated proteins into the String database and filtered for physical interactions of these proteins. Consequently, we identified a core set of ribosomal proteins (RPs), including RPS21, RPS25, RPL35A, RPL37A, RPL6, and LYAR, that is downregulated by TMP269 treatment (Figure 2F).

**Figure 3:**
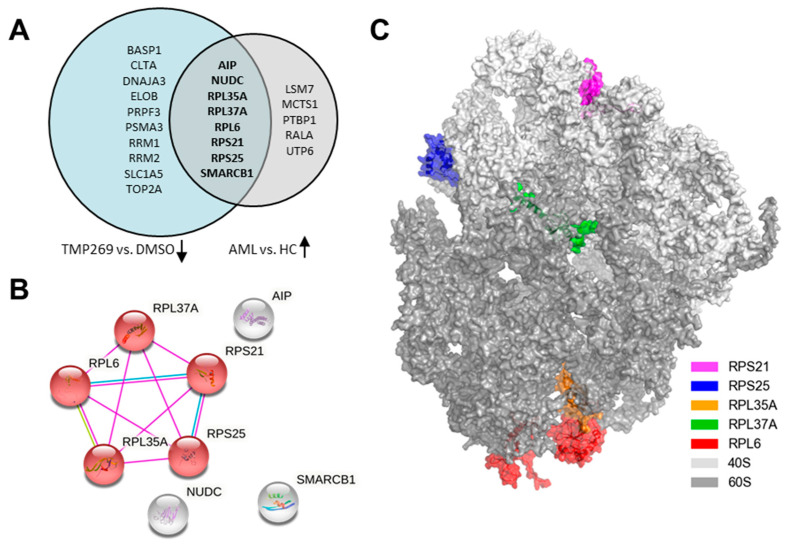
Comparative bioinformatic analysis with the MILE study (Gene Expression Omnibus—GEO: GSE13159). TMP269 treatment downregulates ribosomal proteins which are increased in AML patients at the gene expression level. (**A**) Venn Diagram showing the intersection of 8 proteins downregulated by TMP269 (TMP269 vs. DMSO) which are overexpressed at the gene expression level in AML patients (AML vs. HC). (**B**) String analysis showing physical interactions of overlapping proteins/genes from the intersection in A. Red are proteins involved in the KEGG pathway for *Ribosome*. (**C**) 3D structure of the ribosome showing the large and small ribosomal subunits and the location of the proteins RPS21, RPS25, RPL35A, RPL37A, and RPL6.

Next, we performed a comparative bioinformatic analysis of data from the above-mentioned MILE study and analyzed how many of the 44 significantly differentially expressed proteins are also differentially expressed at the transcriptional level in AML patients compared to healthy controls (Figure 3). We first focused on the proteins downregulated by TMP269 treatment and analyzed whether their respective genes are significantly overexpressed in AML patients. In this comparative analysis, we excluded all genes from the MILE study whose sample size was less than or equal to 460 for AML patients and less than or equal to 68 for healthy controls. Strikingly, we identified eight genes/proteins (*AIP*, *NUDC*, *RPL35A*, *RPL37A*, *RPL6*, *RPS21*, *RPS25,* and *SMARCB1*) that are overexpressed at the transcriptional level in AML patients but downregulated at the protein level upon TMP269 treatment in MOLM-13 cells (Figure 3A). Vice versa, we observed six overlapping genes/proteins (*ANP32E*, *ANXA5*, *NUCB2*, *PSMB3*, *RER1*, and *TPD52*) that are upregulated upon TMP269 treatment in MOLM-13 cells but are expressed at lower transcriptional levels in AML patients (Supplementary Figure S1B). String database analysis of the eight genes/proteins that are overexpressed in AML patients but downregulated upon TMP269 treatment revealed a similar core set of RPs including RPS21, RPS25, RPL35A, RPL37A, and RPL6, all of which are part of the KEGG pathway for *Ribosome* (Figure 3B, red). 3D structure analysis of the ribosome (PDB ID: 4V6X) further defined the location of these proteins within the ribosome. Whereas RPS21 and RPS25 are part of the 40S ribosomal subunit, RPL35A, RPL37A, and RPL6 are constituents of the large 60S ribosomal subunit (Figure 3C). A complete comparison of the proteomic data with the data from the MILE study and the significantly differentially expressed genes in AML patients are presented in Supplementary Data File S1.

Our data clearly demonstrate that the treatment of MOLM-13 cells with the class IIA HDAC inhibitor TMP269 downregulates RPs which are overexpressed at the transcriptional level in AML patients.

### 3.3. Inhibition of Class IIA HDACs by TMP269 Dampens AML Cell Growth and Reduces Cell Proliferation in a Concentration-Dependent Manner

Having shown that TMP269 treatment downregulates RPs, we next evaluated the cellular consequences of TMP269 treatment on AML cells. RPs are essential components of cytosolic ribosomes, involved in ribosome biogenesis and protein synthesis, but RPs also have several extra-ribosomal functions implicated in tumorigenesis. For example, RPs can impact oncogenic pathways involved in cell proliferation, cell survival, cell-cycle progression, apoptosis, glycolysis, and metastasis in a variety of cancer types [52].

**Figure 4:**
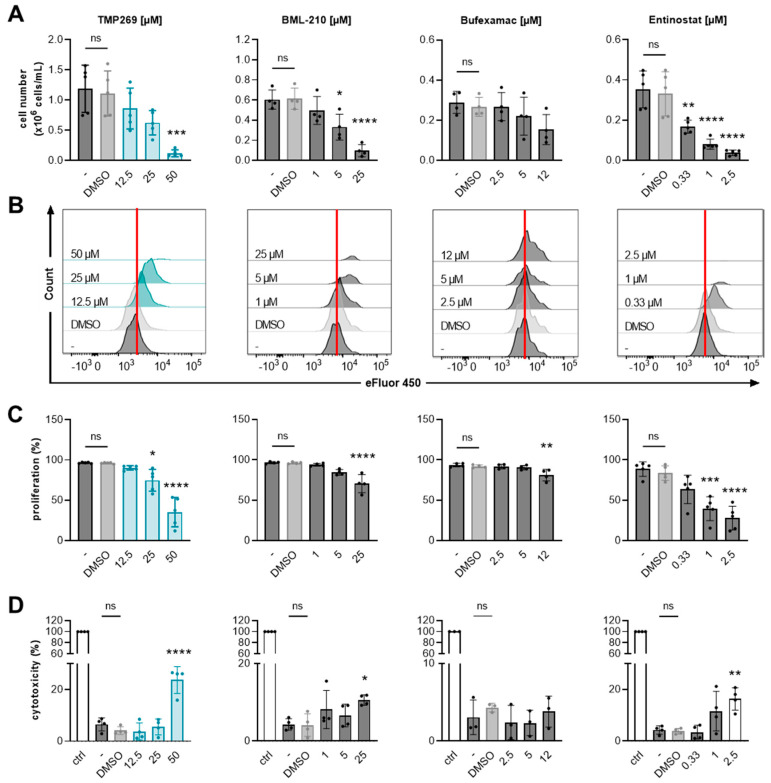
Analysis of AML cell growth and cell proliferation upon treatment with different HDAC inhibitors. TMP269 reduces cell numbers and proliferation of AML cells. MOLM-13 cell numbers, cell proliferation, and cell cytotoxicity were analyzed upon treatment with the indicated concentrations [µM] of class IIA HDAC inhibitors TMP269 and BML-210, class IIB HDAC inhibitor bufexamac, and class I HDAC inhibitor entinostat (MS-275) for 48 h. (**A**) Cell numbers were evaluated using a Neubauer chamber (n = 4–5). (**B**,**C**) Proliferation of MOLM-13 cells was analyzed by flow cytometry, using the cell proliferation dye eFluor 450 (n = 4–5). Histograms of one representative out of 4–5 experiments are shown. The vertical red lines indicate proliferating cells in the DMSO control. (**D**) Cytotoxicity was measured by lactate dehydrogenase (LDH) assay (n = 3–4). Data represent mean ± SD and were statistically analyzed by one-way analysis of variance (ANOVA) with Tukey’s post-hoc test, * *p* ≤ 0.05, ** *p* ≤ 0.01, *** *p* ≤ 0.001, **** *p* ≤ 0.0001. Stars indicate statistical significance compared to the DMSO treated group. ns = not significant. ctrl = control, cells lysed with 0.10% Triton X-100. - = uninduced, untreated cells. DMSO = solvent control, 0.1% DMSO as final concentration.

Initially, we investigated whether TMP269 and subsequent downregulation of RPs has anti-proliferative effects on AML cells in vitro. Additionally, we compared TMP269 to another selective class IIA HDAC inhibitor, BML-210 [53,54,55,56], to the class IIB inhibitor bufexamac [55], and to the well-studied class I HDAC inhibitor MS-275 [48,57,58,59] (Figure 4). We analyzed the effects of these different HDAC inhibitors on cell numbers and cell proliferation by treating MOLM-13 cells with increasing concentrations of the drugs (class IIA HDAC: TMP269 = 12.5–50 µM, BML-210 = 1–25 µM; class IIB HDAC: bufexamac = 2.5–12 µM; class I HDAC: MS-275 (entinostat) = 0.33–2.5 µM). Cell numbers were analyzed after 48 h by manual cell counting, and cell proliferation was analyzed by flow cytometry. The gating strategy for the cell proliferation assay is shown in Supplementary Figure S2A.

TMP269 treatment reduced MOLM-13 cell numbers in a concentration-dependent manner (Figure 4A) and significantly reduced cell proliferation at a concentration of 25 µM compared to the DMSO-treated control (Figure 4B,C). TMP269 treatment at a 25 µM concentration resulted in significantly increased H3K9 acetylation compared to DMSO after 24 h of treatment (Supplementary Figure S1C). We further tested different concentrations of TMP269 for direct cytotoxic effects in our in vitro cell model system and began to observe cytotoxic effects, in terms of lactate dehydrogenase (LDH) release, at 50 µM. Concentrations of 12.5 to 25 µM were well-tolerated by MOLM-13 cells (Figure 4D).

It has already been demonstrated that the class IIA HDAC inhibitor BML210 inhibits cell growth of AML cell lines by promoting apoptosis and cell-cycle arrest in a dose- and time-dependent manner [54,60]. Accordingly, in our study, we observed a significant reduction in cell numbers (Figure 4A) as well as a reduction in cell proliferation (Figure 4B,C) upon treatment with 5 µM BML-210. BML-210 showed cytotoxic effects at a concentration of 25 µM (Figure 4D).

Additionally, we incubated MOLM-13 cells with the class IIB HDAC inhibitor bufexamac, which has already been investigated as a potential therapeutic agent against breast cancer [61]. However, bufexamac did not alter cell numbers significantly (Figure 4A) and we observed only a small reduction in MOLM-13 cell proliferation at 12 µM (Figure 4B,C). At 12 µM, bufexamac showed no signs of cytotoxicity (Figure 4D). The class I HDAC inhibitor entinostat (MS-275) produced the strongest reduction in cell number (Figure 4A) and cell proliferation (Figure 4B,C) in a concentration-dependent manner. Entinostat showed cytotoxicity at 2.5 µM (Figure 4D).

From these data, we conclude that TMP269 has anti-proliferative effects on AML cells comparable to BML-210 and entinostat treatment. We further validated the cell-growth inhibitory effects of TMP269 in three additional AML cell lines, i.e., MV4-11, HL-60, and OCI-AML, and observed a concentration-dependent reduction in cell numbers for all tested AML cell lines (Supplementary Figure S2B).

These data indicate that the class IIA HDAC inhibitor TMP269 is an interesting epigenetic drug and warrants further testing in new combinatory treatments for AML.

### 3.4. Combination Treatment of TMP269 with Venetoclax Has Additive Apoptotic Effects on AML Cells

**Figure 5:**
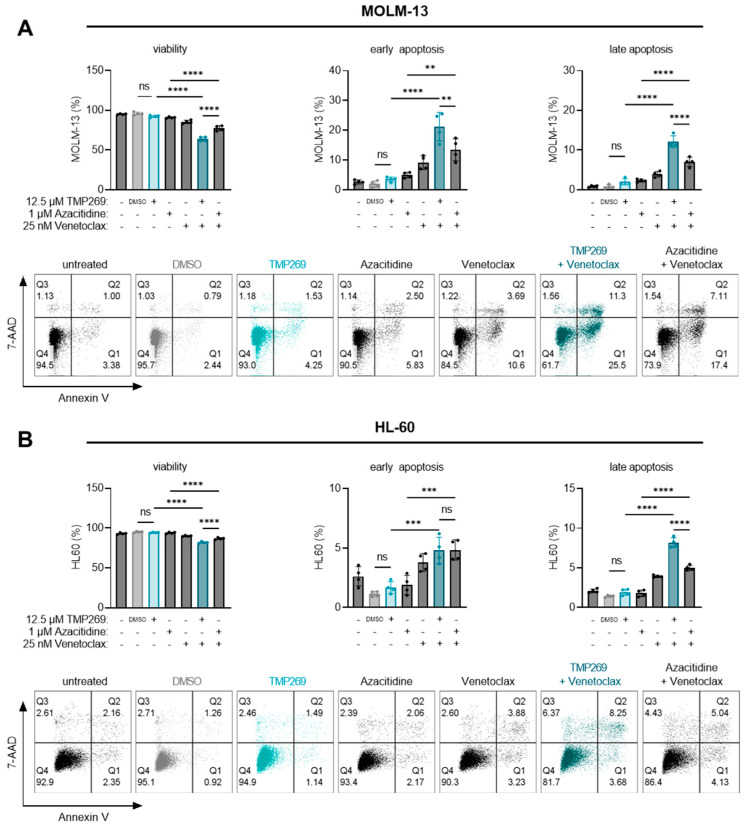
The combination treatment of TMP269 plus venetoclax significantly increases AML cell death compared to azacitidine plus venetoclax. Cells were treated with 12.5 µM TMP269, 1 µM azacitidine, 25 nM venetoclax, or a combination thereof for 24 h. The percentage of MOLM-13 cells (**A**), or HL-60 cells (**B**) that were viable (Annexin V and 7-AAD double negative), early apoptotic (Annexin V positive and 7-AAD negative), or late apoptotic (Annexin V and 7-AAD double positive) was determined by staining for Annexin V and 7-AAD and flow cytometry analysis. Histograms of one representative out of 4 experiments are shown. Dots represent individual experiments (n = 4); bars represent mean ± SD and were statistically analyzed by one-way analysis of variance (ANOVA) with Tukey’s post-hoc test, ** *p* ≤ 0.01, *** *p* ≤ 0.001, **** *p* ≤ 0.0001. ns = not significant. - = uninduced, untreated cells. DMSO = solvent control, 0.1% DMSO as final concentration.

Finally, we assessed whether TMP269 treatment and the observed downregulation of RPs have apoptotic effects or can even boost the apoptotic effects of the BCL-2 inhibitor venetoclax. We also analyzed if the combination of TMP269 with venetoclax is superior to the combination of venetoclax with azacitidine, which is the standard-of-care for AML patients not eligible to receive intensive chemotherapy [8,62]. We analyzed AML cell viability and apoptosis in MOLM-13 and HL-60 cells upon treatment with TMP269, azacitidine, venetoclax, or a combination of either TMP269 and venetoclax or azacitidine and venetoclax (Figure 5). We further treated MOLM-13 cells with BML-210, venetoclax, or a combination of BML-210 with venetoclax (Supplementary Figure S4). For cell death analysis, we used Annexin V and 7-AAD staining and flow cytometry to analyze the percentage of viable (Annexin V and 7-AAD double negative), early apoptotic (Annexin V positive and 7-AAD negative), and late apoptotic cells (Annexin V and 7-AAD double positive). The gating strategy for the cell death analysis is shown in Supplementary Figure S3A.

First, we evaluated the degree of cell death induced by different concentrations of TMP269, azacitidine, venetoclax, or BML-210 alone. TMP269 treatment started to induce early and late apoptosis at 25 and 50 µM concentrations (Supplementary Figure S3B). Azacitidine induced early and late apoptosis at 5 µM (Supplementary Figure S3C). As expected, venetoclax treatment resulted in a dose-dependent increase in early and late apoptosis already at 25 nM (Supplementary Figure S3D). BML-210 induced apoptosis at 25 µM (Supplementary Figure S3E). Next, we tested the additive effects by combining the different treatments. Based on these results and to avoid any masking effects by using too-high concentrations, MOLM-13 or HL-60 cells were treated with either 12.5 µM TMP269, 1 µM azacitidine, 25 nM venetoclax, or a combination thereof for 24 h (Figure 5).

Our results revealed that TMP269 at 12.5 µM alone does not reduce cell viability or induce apoptosis of MOLM-13 (Figure 5A) or HL-60 cells (Figure 5B). However, the viability of MOLM-13 cells (Figure 5A) decreased significantly while the percentage of early and late apoptotic cells increased significantly upon treatment with the combination of TMP269 plus venetoclax or azacitidine plus venetoclax compared to the respective single treatments. The same effects were observed in HL-60 cells (Figure 5B). Unexpectedly, in both AML cell lines, we observed that the combination of TMP269 plus venetoclax at given concentrations resulted in significantly reduced cell viability and significantly higher levels of apoptosis superior to azacitidine plus venetoclax (Figure 5A,B).

We also confirmed this additive apoptosis-inducing effect with the specific class IIA HDAC inhibitor BML-210 in combination with venetoclax (Supplementary Figure S4). Based on our results, we conclude that TMP269 exerts an additive apoptotic effect in combination with venetoclax and is superior to the combination of azacitidine plus venetoclax in AML cells.

In summary, our data demonstrate that specific class I and class II HDAC genes are overexpressed in AML patients. At the molecular level, the class IIA HDAC inhibitor TMP269 downregulates several different RPs. As a cellular consequence, TMP269 treatment has anti-proliferative and additive apoptotic effects when combined with venetoclax in different AML cell lines, suggesting that the combination of TMP269 plus venetoclax may have superior pro-apoptotic effects than the combination of azacitidine and venetoclax, which is already used to treat AML patients.

## 4. Discussion

AML is one of the most common and most aggressive myeloid malignancies, with a very poor 5-year overall survival rate [63]. This leukemia is characterized by altered myeloid progenitor cell proliferation, blocked cell differentiation, and resistance to apoptosis, which result in the replacement of functional blood cells by non-functional leukemic blasts [2,64]. AML is a very heterogenous disease, featuring both genetic mutations and epigenetic alterations as drivers of AML pathophysiology [65]. Most patients are 65 years or older when diagnosed with AML and are therefore not eligible for standard high-dose chemotherapy [66,67]. Consequently, these patients often receive low-dose chemotherapy and/or HMAs, but recently new drugs have been approved for this demographic [68]. For example, the pro-apoptotic agent venetoclax, which is a BCL-2 inhibitor, has been approved since 2018 and applied in combination with HMAs such as azacitidine or decitabine [69]. Despite these advances in AML treatment for older individuals, their overall 5-year survival rate is still only 10–15% [70]. Therefore, there is an urgent need to develop new treatment strategies and to find new combinations of drugs with strong anti-leukemic effects, which are also well-tolerated by older patients.

Cell proliferation and differentiation are tightly regulated processes depending on proper gene expression. Transcription factors regulate a number of hematopoesis-specific genes which are differentially expressed in different types of blood cancer, including AML. Recent evidence suggests that dysregulated epigenetic regulators also contribute to cancer progression [71]. Epigenetic regulators include DNA methyltransferases (DNMTs), histone methyltransferases (HMTs), histone acetyltransferases (HATs), and HDACs, which are enzymes that modulate chromatin structure and thereby fundamentally regulate levels of gene expression [20,72]. In cancer, histone deacetylation results in transcriptional repression and silencing of tumor suppressor genes, thus favoring tumor progression [12,13]. In addition to HMAs such as azacitidine that are already used clinically in some AML patients who are not eligible for intensive chemotherapy [73], new HDAC inhibitors are currently under clinical development [20,74].

Altered expression levels of HDAC and related genes have been reported for different cancer types, including prostate cancer, liver cancer, colon cancer, breast cancer, and neuroblastoma [12]. However, in AML patients, altered expression levels of class I or class II HDACs are poorly described [75] and a detailed screen for differential gene expression of all classes of HDAC genes and their expression profiles in AML patients has not been reported so far.

Our work complements the existing data on dysregulated HDAC gene expression in AML patients. We screened the publicly available database from the MILE study for gene expression levels of all classes of HDAC genes in AML patients and compared them to healthy controls. We found specifically increased class I (*HDAC1*, *HDAC2*) and class II (*HDAC5*, *HDAC7*, *HDAC9*, *HDAC6*, *HDAC10*) HDAC gene expression in AML patients compared to healthy controls and also confirmed the expression of these genes in various AML cell lines, representative of different AML subtypes.

While considerable data are available on the beneficial effects of pan-HDAC inhibitors [20], less is known about the molecular and therapeutic function of specific class IIA inhibitors [76]. Here, we specifically investigated the class IIA HDAC inhibitor TMP269 and its effects on the cellular proteome, cell proliferation, and apoptosis in different AML cell lines. While data on TMP269 in the context of AML are scarce, TMP269 was pre-clinically tested in models for cardiovascular disease [30], urothelial carcinoma [51], and multiple myeloma [50]. In the latter study, TMP269 was reported to have modest growth inhibitory effects and to enhance ER stress-mediated apoptosis in multiple myeloma cell lines in combination with the protease inhibitor carfilzomib [49,50]. Kikuchi et al. showed that TMP269 enhances the pro-apoptosis activities of carfilzomib [50]. Likewise, we observed that TMP269 treatment in combination with venetoclax results in increased apoptosis rates. When used as a monotherapy, TMP269 did not induce apoptosis in AML cells at a concentration of 12.5 µM. Similar sensitizing effects are observed in patients who received the HMA azacitidine in combination with venetoclax. It was shown that azacitidine sensitizes AML cells for venetoclax-mediated apoptosis by inducing the pro-apoptotic molecule NOXA [77]. Similarly, we hypothesize that TMP269 treatment makes AML cells more vulnerable to venetoclax treatment, potentially by the downregulation of ribosomal proteins (RPs). The detailed mechanisms of how TMP269 makes AML cells more vulnerable to apoptosis need to be further investigated.

Different types of HDAC inhibitors directly interfere with the active site of HDACs and, as a result, block deacetylation and foster acetylation of histones or non-histone related proteins [78]. With our proteomic analysis and comparative bioinformatic analysis on TMP269-treated MOLM-13 cells, we identified a core set of downregulated proteins, many of them RPs. Most interestingly, this set of proteins is also upregulated at the gene expression level in AML patients. RPs are not only important components of cytosolic ribosomes, they are also implicated in tumorigenesis via extra-ribosomal functions that interfere with a variety of oncogenic pathways involved in cell proliferation, cell survival, cell-cycle progression, apoptosis, glycolysis, or metastasis. For example, RPs can activate p53-dependent or -independent pathways, resulting in cell-cycle arrest and apoptosis [79]. Thereby, RPs contribute to cell transformation and are frequently dysregulated in a variety of different cancer types [52]. Defects in RPs result in ribosomopathies, and indeed, patients with ribosomopathies are at a higher risk of developing cancer later in life [79]. How RP dysregulation may impact AML is completely unknown.

Our proteomic analysis of TMP269-treated MOLM-13 cells revealed downregulation of the proteins LYAR (highest fold change) and RPL6 (most significant). LYAR is a cell growth-regulating protein and part of the 60S ribosomal subunit that is described to control protein translation [80]. In colorectal cancer, LYAR was shown to promote cancer progression [81]. Consistent with its nucleolar localization, LYAR is known to participate in the regulation of ribosomal gene transcription, rRNA processing, and ribosome biogenesis. LYAR is highly expressed in tumor cells and embryonic stem cells, which are cell types that increase the production of ribosomal proteins in order to support high rates of cell proliferation [82,83]. Overexpression of LYAR was shown to increase cell proliferation without altering the expression of the proto-oncogene c-Myc or the tumor suppressor p53 [83], and it enhances the proliferation and survival of neuroblastoma cells [84]. In non-small-cell lung cancer, expression of LYAR is associated with poor prognosis [85]. The function of LYAR in the context of AML is so far unknown. However, the results of the above-mentioned studies are in line with our own data presented here, as we observed the downregulation of LYAR in AML cells upon treatment with TMP269.

RPL6 is the most significantly downregulated protein in our proteomic analysis. RPL6 is a component of the large ribosomal subunit (60S) [86]. Ribosome biogenesis is essential for cell growth and cell proliferation. In the context of cancer, it was already shown that the expression levels of RPL6 are altered and that RPL6 monitors the level of tumor suppressor p53 in response to ribosomal stress [87]. In multidrug-resistant gastric cancer cells, RPL6 is upregulated and RPL6 overexpression correlates with lower overall survival in cancer patients [88]. In addition, in 2011, Wu et al. showed that the genetic downregulation of RPL6 in gastric cancer cells reduced its colony-forming ability in vitro, reduced cancer growth in vivo, and stopped cell-cycle progression via the downregulation of cyclin E [88]. Moreover, in lung cancer cells, downregulation of RPL6 inhibits cancer cell proliferation and migration and promotes cell apoptosis [89]. Furthermore, Zhang et al. showed that knockdown of RPL6 in lung cancer cells is accompanied by downregulation of BCL-2 and AKT signaling activators such as p-AKT and p-S6. Thereby, RPL6 downregulation leads to increased protein levels of pro-apoptotic molecules such as cleaved caspase-3 and BAX [89]. However, the role and function of RPL6 in AML are still largely unknown. In the present work, we demonstrated a link between RPL6 and AML, as the *RPL6* gene is overexpressed in AML patients compared to healthy controls. Furthermore, the downregulation of RPL6 protein levels induced by TMP269 treatment is associated with increased AML cell apoptosis when the cells are additionally treated with venetoclax.

Our String analysis revealed that RPL6 directly interacts with other RPL proteins such as RPL35A, RPL37A, RPS21, and RPS25, which are also significantly downregulated by TMP269 treatment and upregulated at the gene expression level in AML patients.

RPS25 has recently been shown to play an essential role in the cap-independent initiation of translation, which suggests that RPS25 has an important general function in regulating protein translation [90]. The *RPS25* gene was demonstrated to be overexpressed in human leukemia cells which exhibit chemotherapy resistance to adriamycin [91]. Furthermore, RPS25 was suggested to play a role in apoptosis [92] and cell-cycle arrest [93]. However, little is known about the role of RPS25 in the context of AML.

Another candidate to be investigated based on our proteome analysis is RPL35A, which was also downregulated upon TMP269 treatment but is upregulated at the transcriptional level in AML patients. In gastric cancer, *RPL35A* knockdown inhibited cell proliferation and migration, promoted apoptosis, and suppressed tumor growth [94]. Furthermore, overexpression of RPL35A was shown to inhibit cell death without affecting the levels of anti-apoptotic proteins such as BCL-2 and BCL-xL [95]. This indicates that RPL35A might also have direct anti-apoptotic, extra-ribosomal functions in cancer cells.

## 5. Conclusions

Although a significant proportion of AML patients can now be cured, studies on newer treatment options such as the BCL-2 inhibitor venetoclax revealed that patients can become resistant to this drug, resulting in lower survival rates [96]. Finding new drugs that might be combined with already existing drugs is key for increasing the overall survival of AML patients. Here, we showed that specific class I and class II HDAC genes are significantly overexpressed in AML patients compared to healthy controls and that combinatory treatment of AML cell lines with the class IIA HDAC inhibitor TMP269 and venetoclax leads to increased induction of cell apoptosis. The combination of TMP269 plus venetoclax might therefore be a new treatment option for AML patients.

## Data Availability

The raw data supporting the conclusions of this article will be made available by the authors upon reasonable request.

## Supplementary Materials

The following supporting information can be downloaded at: https://www.mdpi.com/article/10.3390/cancers15041039/s1, Supplementary Materials and Methods for Proteomics; Supplementary Figure S1: HDAC gene expression in AML patients, comparative bioinformatic analysis with the MILE study (Gene Expression Omnibus—GEO: GSE13159) and H3K9 acetylation analysis; Supplementary Figure S2: Representative FACS plots for the gating strategy in the proliferation assay and treatment of different AML cell lines with increasing concentrations of TMP269; Supplementary Figure S3: Gating strategy for the apoptosis assay, and treatment of MOLM-13 cells with increasing concentrations of TMP269, azacitidine, venetoclax. and BML-210 for cell death analysis; Supplemenary Figure S4: The combination treatment of BML-210 plus venetoclax significantly increases AML cell death; Supplementary Data File S1.
