# Supplementary material for: The Class IIA Histone Deacetylase (HDAC) Inhibitor TMP269 Downregulates Ribosomal Proteins and Has Anti-Proliferative and Pro-Apoptotic Effects on AML Cells
Source: Cancers (Basel). 2023 Feb 7;15(4):1039. doi: 10.3390/cancers15041039 (PMC9953883; doi:10.3390/cancers15041039)
Supplement: Supplementary file 1 [file cancers-15-01039-s001.zip › Supplementary data_6.2.23.pdf]

cancers

MDPI

Article

The Class IIA histone deacetylase (HDAC) inhibitor TMP269 downregulates ribosomal proteins and has anti-proliferative and pro-apoptotic effects on AML cells

Laura Urwanisch[1,2,†], Michael Stefan Unger[1,2,†], Helene Sieberer[1,2], Hieu-Hoa Dang[1,2], Theresa Neuper[1,2], Christof Regl[1,2], Julia Vetter[3], Susanne Schaller[3], Stephan M Winkler[3], Emanuela Kerschbamer[4], Christian X Weichenberger[4], Peter W Krenn[1,2], Michela Luciano[1,2], Lisa Pleyer[2,5,6], Richard Greil[2,5,6], Christian G. Huber[1,2], Fritz Aberger[1,2], Jutta Horejs-Hoeck[1,2,*]

1. Supplementary Materials and Methods for Proteomics

1.1. Chemicals

Ultrapure water for solutions was produced in-house with a MilliQ® Integral 3 instrument (Millipore, Billerica, MA, USA). Acetonitrile (ACN, ≥ 99.9%) and methanol were acquired from VWR International (Vienna, Austria). Triethylammonium bicarbonate buffer (TEAB, pH 8.5 ± 0.1, 1 mol.L-1), sodium dodecyl sulfate (SDS, ≥ 99.5%), tris(2-carboxyethyl)phosphine-hydrochloride (TCEP, ≥ 98.0%), iodoacetamide (IAA, ≥ 99.0%), formic acid (FA, 98.0-100%) and cOmplete Protease Inhibitor Cocktail tablets (Roche) were obtained from Sigma-Aldrich (Vienna, Austria). Ortho-phosphoric acid (85%) was from Merck (Darmstadt, Germany). Trypsin (sequencing grade modified, porcine) was purchased from Promega (Madison, WI, USA).

1.2. Sample preparation

Samples were prepared by suspension trapping utilizing S-Trap micro columns from Protifi (NY, USA) with minor adaptions to the manufacturer's instructions. Cells were lysed in 5% SDS in 50 mmol.L-1 TEAB (pH 8.50) at 95°C for 5 min followed by sonication in a Bioruptor device (Diagenode, Liège, Belgium) for 10 min. After a 10 sec centrifugation step at 13,000 g, protein content in the supernatant was analyzed by a Pierce BCA Protein assay kit (Thermo Fisher Scientific, Vienna, Austria) according to the manufacturer's instructions. An amount of 50 µg protein per sample was further treated with 5 mmol.L-1 TCEP at 55°C for 15 min to reduce disulfide bonds, followed by alkylation of cysteine residues by addition of IAA to a concentration 40 mmol.L-1 and incubation at 22°C in the dark for 15 min. Subsequently the samples were acidified to pH ≤1 with ortho-phosphoric acid before purification by the S-Trap micro columns. For proteolysis, trypsin was added to give an enzyme to protein ratio of 1:10 (w/w) followed by overnight incubation at 37°C. The obtained peptides were eluted from the S-trap columns, dried at 45°C in a vacuum centrifuge, and resuspended in 100 mmol.L-1 TEAB (pH 8.5).

1.3. High-performance liquid chromatography coupled to mass spectrometry

Chromatographic separation of 1 µg of each sample was carried out on an UltiMate™ 3000 RSLCnano System (Thermo Fisher Scientific, Germering, Germany), employing a µPAC™ C18 separation column (2000 x 0.040 mm i.d.) from PharmaFluidics, Ghent, Belgium. For separation of the peptides, 0.10% aqueous FA (solvent A) and 0.10% FA in ACN (solvent B) were pumped at a flow rate of 300 nL.min-1 in the following order: 1.0% B for 10.0 min, a linear gradient from 1.0-25.0% B in 170 min and a second linear gradient from 25.0-45.0% B in 20.0 min. This was followed by flushing with 80.0% B for 10 min and column re-equilibration with 1.0% B for 60 min. The column temperature was kept constant at 50°C.

The nanoHPLC system was hyphenated to a Q Exactive™ Plus Hybrid Quadrupole-Orbitrap™ mass spectrometer via a Nanospray Flex™ ion source (both from Thermo

Fisher Scientific, Bremen, Germany). The source was equipped with a SilicaTip emitter with 360 µm o.d., 20 µm i.d. and a tip i.d. of 10 µm purchased from CoAnn Technologies Inc. (Richland, WA, USA). The spray voltage was set to 1.5 kV, S-lens RF level to 55.0 and capillary temperature to 320 °C. Each scan cycle consisted of a full scan at a scan range of *m*/*z* 350–2,000 and a resolution setting of 70,000 at *m*/*z* 200, followed by 15 data-dependent higher-energy collisional dissociation (HCD) scans in a 2.0 *m*/*z* isolation window at 32% normalized collision energy at a resolution setting of 17,500 at *m*/*z* 200. For the full scan the automatic gain control (AGC) target was set to 3e6 charges with a maximum injection time of 100 ms; for the HCD scans the AGC target was 1e5 charges with a maximum injection time of 100 ms. Already fragmented precursor ions were excluded for 30 seconds. Data acquisition was conducted using Thermo Scientific™ Chromeleon™ 7.2 CDS (Thermo Fisher Scientific, Germering, Germany).

1.4. Data evaluation

The peptide spectra were analyzed by utilizing MaxQuant 2.0.1.0 [33] and the default settings for label-free quantification (LFQ). For protein identification, a database from the Uniprot consortium [34] including only reviewed Swiss-Prot entries for *Homo sapiens* (Human) from 03.02.2022 was used, applying a 1% false discovery rate and a reversed sequence decoy database. The obtained protein groups were further processed using the Perseus software platform [35]. The identified protein groups were filtered to remove decoy hits as well as proteins that were only identified by site. Next, the LFQ intensities were log2-transformed and normalized by subtraction of the median.

2. Supplementary Figures

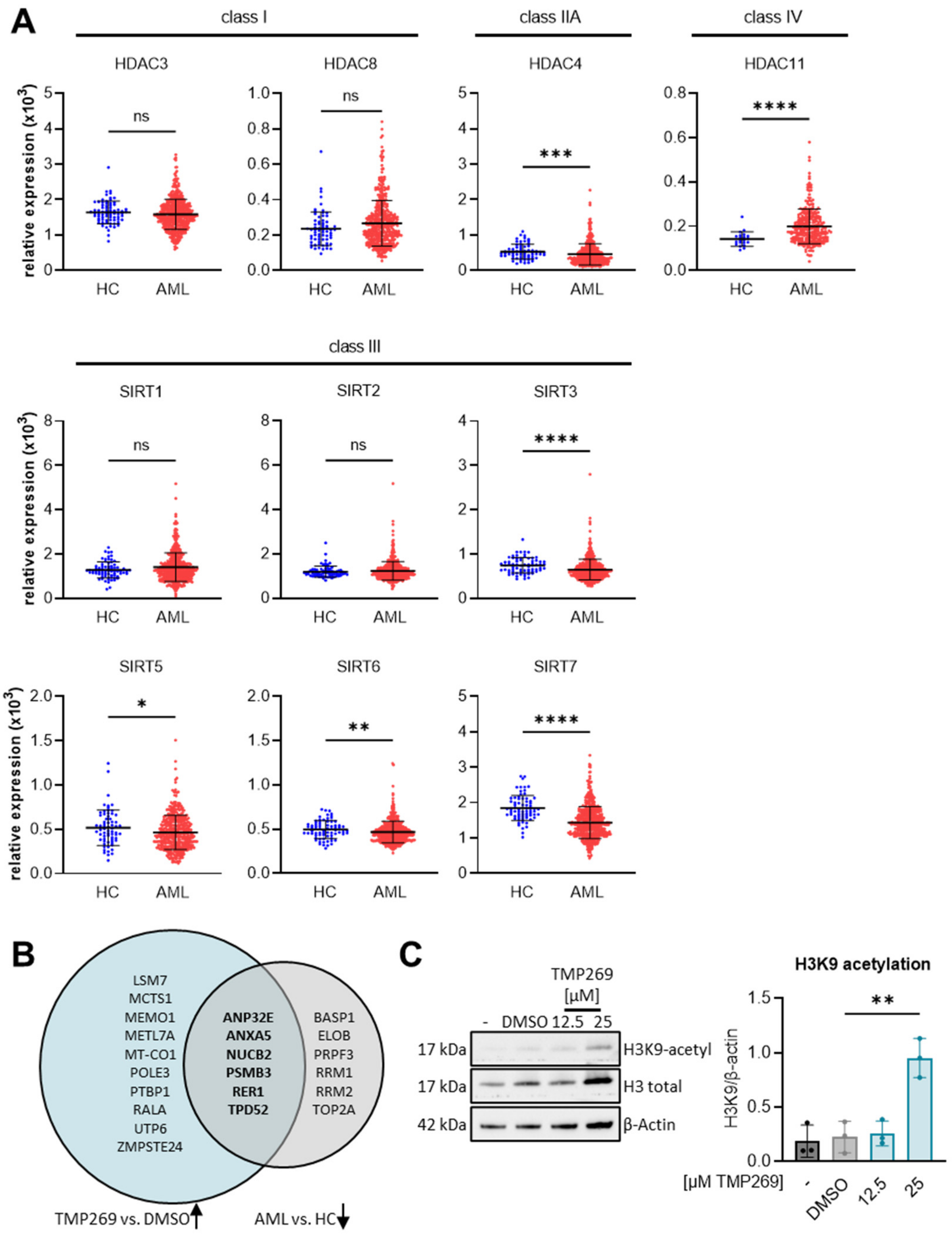

Supplementary Figure S1: HDAC gene expression in AML patients, comparative bioinformatic analysis with the MILE study (Gene Expression Omnibus - GEO: GSE13159) and H3K9 acetylation analysis. (A) Significantly reduced *HDAC4*, *SIRT3*, *SIRT5*, *SIRT6* and *SIRT7* gene expression in AML patients compared to healthy controls (HC). *HDAC11* was significantly overexpressed in AML patients. The dataset was imported using GEOparse from Python. Data are mean ± SD and were statistically analyzed by two-tailed Mann-Whitney U test, *p ≤ 0.05, **p ≤ 0.01, ***p ≤ 0.001, ****p ≤ 0.0001. **(B)** Venn diagram showing the intersection of 6 proteins upregulated by TMP269 treatment in MOLM-13 cells (TMP269 vs. DMSO) which are downregulated at the gene expression level in AML patients (AML vs. HC). **(C)** Western blot analysis for H3K9 acetylation, H3 total and β-actin in MOLM-13 cells treated with 12.5 and 25 µM TMP269 for 24 hours. H3K9 acetylation was significantly increased in cells treated with 25 µM TMP269. Representative blot out of 3 replicates (n=3). Data are mean ± SD and were statistically analyzed by one-way analysis of variance (ANOVA) with Tukey's post-hoc test, **p ≤ 0.01. ns = not significant. - = uninduced, untreated cells. DMSO = solvent control, 0.1% DMSO as final concentration.

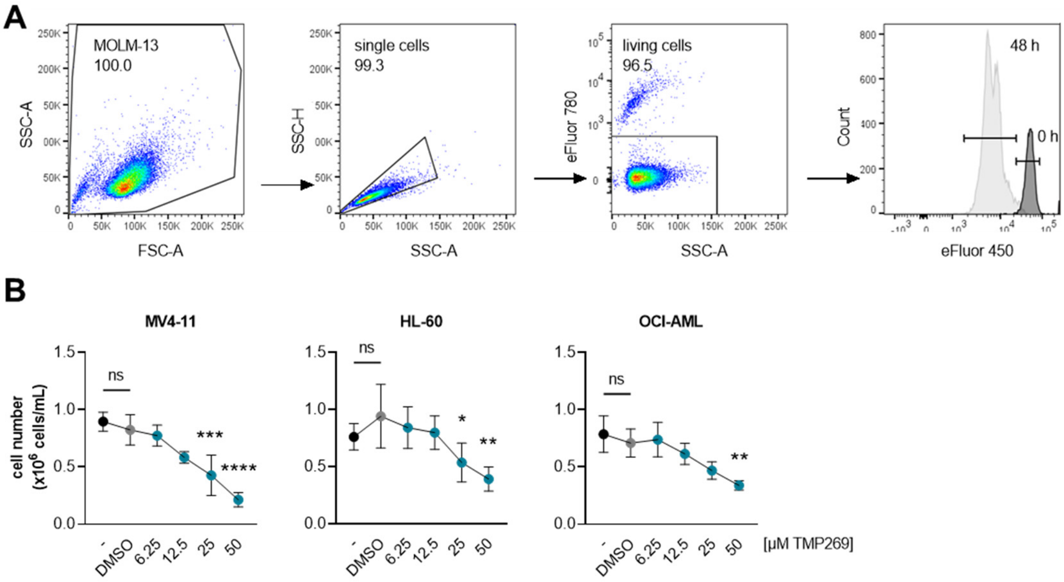

Supplementary Figure S2: Representative FACS plots for the gating strategy in the proliferation assay and treatment of different AML cell lines with increasing concentrations of TMP269. (A) After 48 hours of incubation with the cell proliferation dye, live single MOLM-13 cells were gated for eFluor 450 fluorescence signal. To mark non-proliferating cells, MOLM-13 cells were immediately stained (0 hours) and analyzed for eFluor 450 fluorescence signal (non-proliferation control). Proliferating cells can be distinguished from non-proliferating cells upon reduction of eFluor 450 fluorescence signal. **(B)** Cell numbers were assessed upon treatment with the indicated concentrations of TMP269 after 48 hours in different AML cell lines (MV4-11, HL-60, OCI-AML) by manual cell counting using a Neubauer chamber (n=4). TMP269 treatment significantly reduced cell numbers in a concentration-dependent manner in all tested AML cell lines. Data are mean ± SD and were statistically analyzed by one-way analysis of variance (ANOVA) with Tukey's post-hoc test, *p ≤ 0.05, **p ≤ 0.01, ***p ≤ 0.001, **** p ≤ 0.0001. Stars indicate statistical significance compared to the DMSO-treated group. ns = not significant. - = uninduced, untreated cells. DMSO = solvent control, 0.1% DMSO as final concentration.

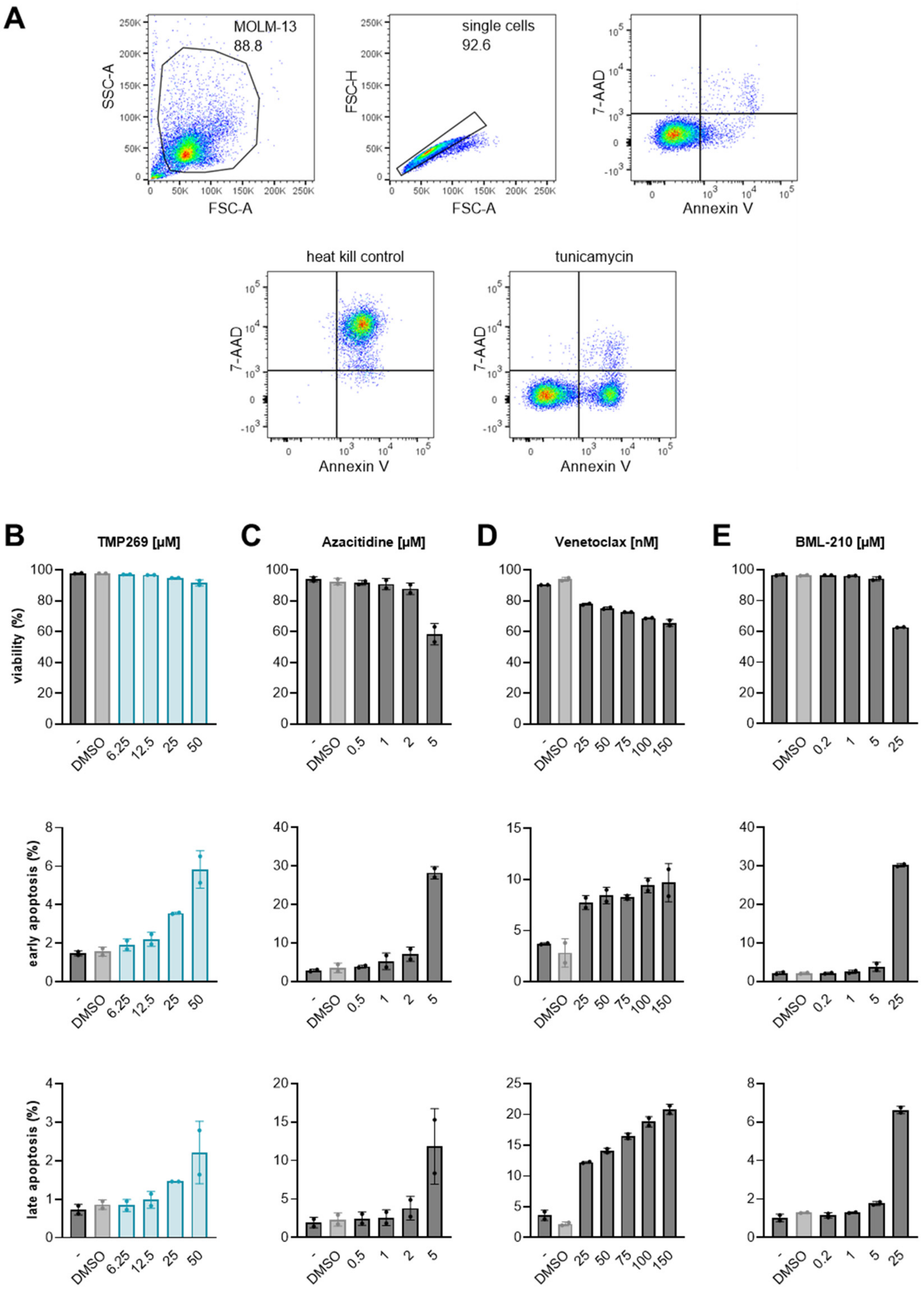

Supplementary Figure S3: Gating strategy for the apoptosis assay, and treatment of MOLM-13 cells with increasing concentrations of TMP269, azacitidine, venetoclax and BML-210 for cell death analysis. (A) Representative FACS plots showing gating strategy for Annexin V and 7-AAD staining in the apoptosis assay. Single MOLM-13 cells were gated for percentages of viable (Annexin V and 7-AAD negative), early apoptotic (Annexin V positive and 7-AAD negative), or late apoptotic MOLM-13 cells (Annexin V and 7-AAD positive). For positive control of early apoptosis, cells were treated with 10 µg/mL tunicamycin; for late apoptosis, the cells were heat-killed. MOLM-13 cells were treated with 6.25-50 µM TMP269 **(B)**, 0.5-5 µM azacitidine **(C),** 25-150 nM venetoclax **(D)**, or 0.2-25 µM BML-210 **(E)** for 24 hours. Data are mean ± SD (n=2). - = uninduced, untreated cells. DMSO = solvent control, 0.1% DMSO as final concentration.

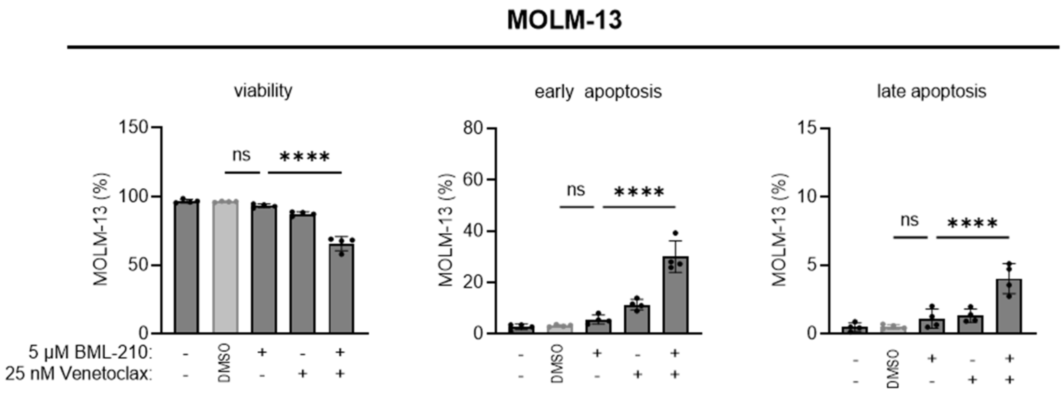

Supplementary Figure S4: The combination treatment of BML-210 plus venetoclax significantly increases AML cell death. MOLM-13 cells were treated with 5 µM BML-210, 25 nM venetoclax or a combination thereof for 24 hours. The percentage of MOLM-13 cells that were viable (Annexin V and 7-AAD double negative), early apoptotic (Annexin V positive and 7-AAD negative), or late apoptotic (Annexin V and 7-AAD double positive) was determined by staining for Annexin V and 7-AAD and flow cytometry analysis. Dots represent individual experiments (n=4), bars represent mean ± SD and were statistically analyzed by one-way analysis of variance (ANOVA) with Tukey's post-hoc test **** $p \leq 0.0001$. ns = not significant. - = uninduced, untreated cells. DMSO = solvent control, 0.1% DMSO as final concentration.
